# Efficacy and safety of thoracoscopic pericardial window in patients with pericardial effusions: a single-center case series

**DOI:** 10.1186/s13019-016-0488-x

**Published:** 2016-06-13

**Authors:** Ichiro Sakanoue, Hiroshi Hamakawa, Yu Okubo, Kazuhiro Minami, Ei Miyamoto, Yu Shomura, Yutaka Takahashi

**Affiliations:** Thoracic Surgery, Kobe City Medical Center General Hospital, 2-1-1 Minatojimaminami-machi, Chuo-ku, Kobe, 650-0047 Japan; Thoracic Surgery, Graduate School of Medicine, Kyoto University Hospital, 54 Kawaracho, Shogoin, Sakyo-ku, Kyoto, 606-8507 Japan; Cardiovascular and Thoracic Surgery, Shingu Municipal Medical Center, 18-7 Hachibuse, Shingu, Wakayama 647-0072 Japan

**Keywords:** Pericardial effusion, Pericardial window, Thoracoscopic surgery

## Abstract

**Background:**

Pericardial effusion (PE) is a common finding in patients who had chronic cardiac failure, who had undergone cardiac surgery, or who had certain other benign and malignant diseases. PE ranges in severity from mild, asymptomatic effusions to cardiac tamponade. Although a thoracoscopic pericardial window (TPW) is a minimally invasive surgical option for patients with PE, there are few published data regarding the outcomes of TPW for PE. We investigated the contribution of the TPW to the treatment of PEs that are recurrent or difficult to drain percutaneously.

**Methods:**

We conducted a retrospective chart review of the indications for TPW that included data on preoperative, intraoperative, and postoperative variables; morbidity; recurrence; and survival. Fourteen consecutive patients with PE that was recurrent or difficult to drain percutaneously and who underwent treatment with a TPW were enrolled in this study. Trocars for passage of the thoracoscope and surgical instruments were introduced through two or three incisions. Mini-thoracotomy was also performed in patients with hemopericardium and loculated fibrinous effusions. All patients were evaluated by face-to-face interviews, transthoracic echocardiography (TTE), and chest radiography 3–6 months after the TPW was obtained.

**Results:**

The mean age of the patients was 70 years (range 28–83 years). The operative time was 72.1 ± 29.5 min. Six patients had undergone open heart surgery during the month prior to their presentation with PE. No intraoperative or postoperative complications occurred, although PE had recurred in one patient. Two patients died of malignant disease several months after the TPW. The cardiothoracic ratio (determined on chest radiographs) and the ejection fraction ratio (determined using TTE) had improved at the 3- and 6-month follow-up evaluations (*p* < 0.0001 and *p* = 0.012, respectively). Some patients could discontinue diuretics after the procedure, as assessed by the cardiologist based on symptom alleviation, chest radiography, and TTE findings.

**Conclusions:**

For patients with PEs that are recurrent or difficult to drain percutaneously, TPW is an effective, safe surgical approach in terms of cardiac function and radiological findings.

## Background

Pericardial effusion (PE) is a common finding in patients who had chronic cardiac failure, who had undergone cardiac surgery, or who had certain other benign and malignant diseases. Its severity ranges from mild, asymptomatic effusion to cardiac tamponade [1–3]. Repeated percutaneous pericardiocentesis or temporary pericardial drainage is frequently required. Performance of pericardiocentesis from the parasternal or xiphoid process region is sometimes difficult because of factors such as hepatomegaly and the main location of the PE. If pericardiocentesis is not feasible or fails, creation of a so-called pericardial window should be considered either by conventional heart surgery or video-assisted thoracoscopy [3]. A thoracoscopic pericardial window (TPW), another option for managing PE, requires a surgeon with experience in thoracoscopy. A true window can be created by partial pericardiectomy, creating a passage that presumably allows longer-term drainage into an adjacent space, usually the pleural space [2]. Additionally, the TPW usually enables treatment while establishing a diagnosis by pericardiectomy [4], and it is less invasive than conventional thoracotomy. Despite these advantages, however, there are few published data regarding the outcome of TPW for PEs that are recurrent or difficult to drain. The purpose of this study was to evaluate retrospectively the efficacy and safety of TPW in patients with PE that were recurrent or difficult to drain percutaneously.

## Methods

Data were collected from inpatient records of 14 patients who had undergone TPW procedures for PE that had been diagnosed by transthoracic echocardiography (TTE) from 2010 to 2014 at the Department of Thoracic Surgery of Kobe City Medical Center General Hospital. The institutional review board approved use of these data for research (No. 14043; August 11, 2015). Specific individual consent for the study was waived. All patients had PE that was recurrent or difficult to drain. The propriety of percutaneous drainage before TPW and the necessity of TPW were evaluated by a cardiologist based on clinical symptoms and computed tomography (CT) and TTE findings. Patients with PE due to blunt chest trauma were excluded from this study.

Preoperatively, all patients underwent a blood count, clotting tests, routine biochemical tests, chest radiography, and CT. They then underwent TPW under general anesthesia in a lateral position with single-lung ventilation using a double-lumen endotracheal tube. Trocars for passage of the thoracoscope and surgical instruments were introduced through two or three incisions. A 3- to 4-cm mini-thoracotomy was also performed in patients with hemopericardium and loculated fibrinous effusion. A 5- or 10-mm thoracoscope (HOPKINS II Telescope; Karl Storz, Tuttlingen, Germany) was used for the procedure.

**Fig. 1:**
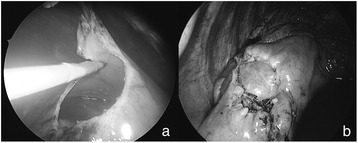
**a** Process of creating a pericardial window with an electric scalpel. **b** Completion of pericardial window, which is 2–4 cm in diameter

A portion of pericardium (2 cm diameter) was resected anterior to the phrenic nerve by a scalpel or scissors (Fig. 1). Any existing PE was removed at the same time. A chest drain was placed in the pleural cavity in all cases and was removed once the daily drainage had decreased to <200 mL. All patients were evaluated in terms of demographic characteristics as well as the nature and amount of effusion, operation time, and treatment results using face-to-face interviews, TTE, and chest radiography 3–6 months after the surgery. Changes in clinical symptoms before and after TPW were obtained during the interviews. Recurrence was defined as an effusion visible on postoperative TTE and requiring further therapy.

Student’s paired *t*-test was performed using EZR software (Saitama Medical Center, Jichi Medical University, Saitama, Japan) to assess the statistical significance of differences. A value of *p* < 0.05 was considered to indicate statistical significance. EZR is a graphic user interface for R software (The R Foundation for Statistical Computing, Vienna, Austria).

## Results

The patients’ clinical data are shown in Table 1. There were eight male and six female patients with a median age of 70 years (range 28–83 years). All of the patients had symptoms, such as fatigue, dyspnea on effort, and/or edema. Six patients had undergone open heart surgery during the month prior to their presentation with PE. The propriety of performing pericardiocentesis before the TPW and the need to create a TPW were evaluated by a cardiologist based on clinical symptoms and CT and TTE findings.

**Table 1:** Preoperative and postoperative characteristics of patients (*n* = 14)

Patient	Age (years), sex	Etiology	Symptoms	Side of surgery	Operation duration (min)	PE volume (mL)	Type of PE
1^a,b^	62 M	After cardiac surgery	Fatigue	Left	141	950	Serous
2	65 F	After cardiac surgery	Fever	Left	102	60	Serous
3^b^	77 M	After aortic surgery	DOE, fatigue	Left	68	230	Bloody
4^a^	76 F	Heart failure	Fatigue, edema	Right	53	350	Serous
5^a,b^	80 F	Heart failure	Dyspnea, edema	Right	31	350	Serous
6^ab^	83 M	Idiopathic	DOE, edema	Left	84	1250	Bloody
7^a^	76 F	Heart failure	Edema, oliguria	Right	37	630	Serous
8	65 M	Malignant disease	DOE, tachycardia	Right	52	600	Bloody
9^a^	78 M	After cardiac surgery	Fatigue, edema	Left	60	N/A	Hematoma
10^b^	61 M	After cardiac surgery	Dyspnea, edema	Left	71	200	Bloody
11^b^	28 F	Malignant disease	Dyspnea, edema	Right	107	230	Bloody
12	65 F	After cardiac surgery	DOE, edema	Left	89	N/A	Hematoma
13^a,b^	70 M	Chronic pericarditis	Edema	Right	75	420	Serous
14^a,b^	74 M	After cardiac surgery	Dyspnea, edema	Left	40	600	Serous
Mean ± SD					72.1 ± 29.5		

*M* male, *F* female, *DOE* dyspnea on effort, *N/A* not available, *PE* pericardial effusion

^a^Pericardiocentesis was performed before creation of the thoracoscopic pericardial window (TPW)

^b^Pleural effusion was performed at the same time as creation of the TPW

Eight patients had undergone pericardiocentesis before TPW. The other six had not because of various anatomical difficulties, which were assessed by the cardiologist. The operative side was the right in six patients and the left in eight. The TPW operation time was 72.1 ± 29.5 min (mean ± SD).

There were no intraoperative or postoperative complications associated with the procedure. The PE recurred following TPW usage in one patient (case 10), who underwent pericardiectomy for chronic constrictive pericarditis 1 month after use of the TPW. Patients 7 and 11 died of malignant diseases 3 and 5 months, respectively, after TPW usage.

**Fig. 2:**
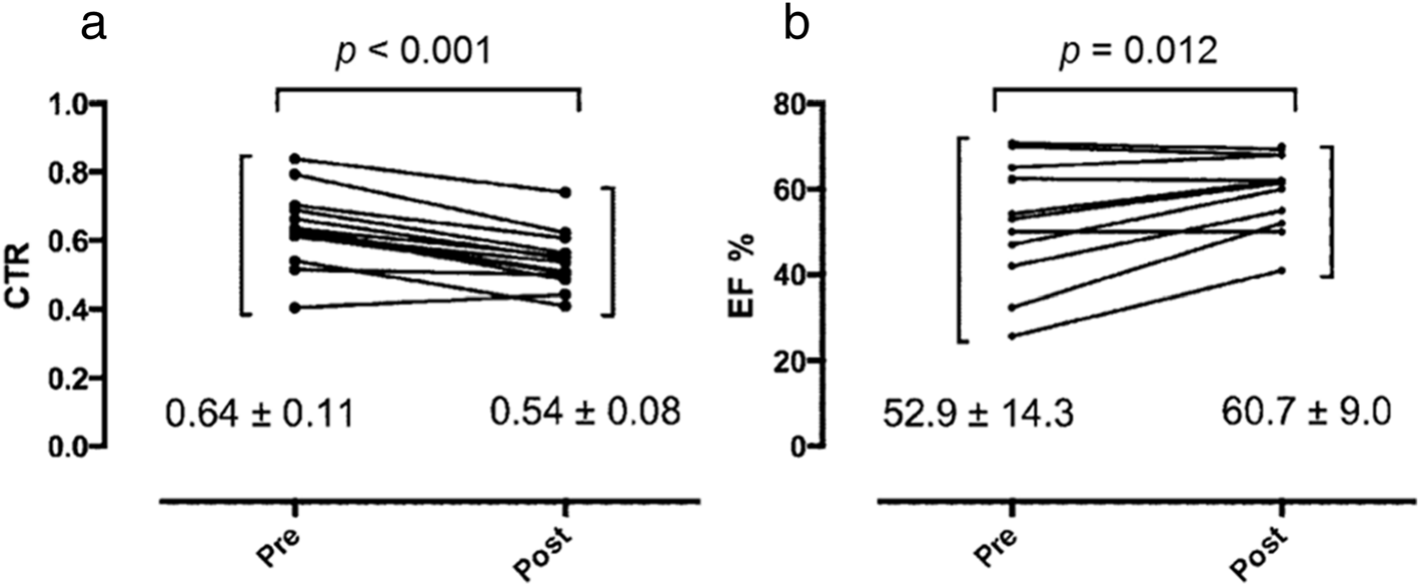
Changes in the cardiothoracic ratio (**a**) and ejection fraction (**b**) before and after creating a thoracoscopic pericardial window (TPW). The data show significant changes after the TPW was created as evaluated by the paired *t*-test (*p* < 0.001 and *p* = 0.012, respectively)

The average cardiothoracic ratio on chest radiography was decreased at 0.098 and the average ejection fraction ratio on TTE was improved by 6.9 % at the 3- to 6-month follow-up evaluation. Both of these changes were statistically significant (*p* < 0.001 and *p =* 0.012, respectively) (Fig. 2). Moreover, some patients were able to discontinue diuretics after the procedure, a decision made by the cardiologist based on alleviation of symptoms, chest radiography, and TTE findings.

## Discussion

Various approaches to the diagnostic and therapeutic assessments of pericardial diseases have been described, including pericardiocentesis, percutaneous catheter drainage, balloon pericardiotomy, subxiphoid pericardial drainage, pericardioperitoneal shunt, subxiphoid pericardial fenestration, and pericardial window creation via anterior thoracotomy or thoracoscopy [5].

Pericardiocentesis is noninvasive and may result in prompt relief for patients with pericardial tamponade. A high complication rate has been reported, however, especially if pericardiocentesis is performed for small, loculated effusions and particularly without echocardiographic visualization [6]. The most serious complications of pericardiocentesis are laceration and perforation of the myocardium and coronary vessels. Patients could also experience air embolism, pneumothorax, arrhythmias (usually vasovagal bradycardia), and puncture of the peritoneal cavity or abdominal viscera [3]. Prolonged catheter drainage is an effective means of preventing fluid reaccumulation, although the mechanism by which it occurs is probably related more to obliteration of the pericardial space after catheter-provoked inflammation than to the fluid drainage itself. This approach is reportedly successful in >70 % of cases, although the duration of effusion control is often not reported [7, 8].

Our study indicates that the TPW for patients with PEs that are recurrent or difficult to drain percutaneously is an effective, safe procedure. Unlike pericardiocentesis, TPW drains PEs into the thoracic cavity through a pericardial window [1, 2, 4]. The effusion is then absorbed through the pleura. Because general anesthesia with single-lung ventilation is necessary to create the TPW, it is not the first choice for managing PE. However, it should be considered as an option in patients who have recurrent PE caused by some inflammatory diseases or chronic heart failure, those who have undergone cardiac surgery, or patients in whom the risk of complications associated with pericardiocentesis is high. TPW allows us easy visualization of the pericardium. The frequency of laceration and peroration of the myocardial structure is expected to be low, especially in patients with PEs that are difficult to drain percutaneously. Celik et al. reported that TPW is also safe and effective for patients with cancer [9].

In our study, two patients for whom TPW was effective died of malignant disease progression within 6 months after TPW usage (Table 1). Hence, the prognosis of any co-morbidities should be taken into account when considering this procedure. Given the poor prognosis of most patients presenting with malignant PE, the primary goals of treatment are to alleviate the symptoms and improve the patient’s quality of life [3].

An additional benefit of the TPW is that the pathology examination of the resected pericardium sometimes reveals the cause of the PE. Robles et al. reported that thoracoscopic management of PEs simply and effectively creates a large pericardial window that drains the effusion and determines its etiology [10]. After TPW creation in some of our patients, the pathological findings of the resected pericardium helped us establish the treatment principles.

Pleural effusion can also be removed during the procedure when needed. Eight patients in our study underwent drainage of pleural effusion at the same time as when the TPW was performed (Table 1). The combined effect of TPW and drainage of pleural effusion might result in a favorable outcome and lower incidence of fluid reaccumulation as a result of improved cardiac function. After TPW creation, only one patient (case 10) experienced recurrence of PE that required an additional procedure or drainage. Although the other patients had a small amount of PE, further therapy was not required. Based on these findings, we believe that pericardial adhesion tends to progress after the TPW, leading to a lower incidence of recurrence.

Subxiphoidal window is an alternative to the TPW, but it can drain PE temporally only while the drainage tube is connected to a closed bag. Several studies of the subxiphoidal window reported complication rates of < 4 % [2, 11, 12], although higher rates have been reported as well [13]. Although six patients in our study had undergone prior open heart surgery, no complications associated with the TPW were observed.

Previous reports have described a low incidence of pericardial tamponade recurrence and the high safety of TPW [2]. Few reports have reported changes in the cardiothoracic ratio and ejection fraction. Based on the fact that these clinical data improved after the TPW was in place in our study, the central venous pressure (CVP) is expected to be a good indicator for assessing the efficacy of the surgery. In our study, however, there were too few patients who required catheterization to justify measuring the CVP preoperatively. Our data suggest that the TPW might be considered a more proactive procedure depending on the patient’s clinical situation, even when the patient has undergone cardiac surgery. Additionally, the risk of effusion recurrence is lower than that associated with other procedures.

Several recent studies have shown that single-incision surgery with thoracoscopy is feasible and less invasive than other methods for treating thoracic diseases [14, 15]. Similar studies should be performed to clarify the efficacy and safety of single-incision surgery with thoracoscopy for treating PE.

This study has several limitations. First, because it was a retrospective study, there was potential patient selection bias owing to the wide variety of etiological agents. Evidence that the TPW is the only, or best, way to avoid repeated pericardiocentesis is inconclusive because the effects of diuretics and other medications cannot be eliminated. Second, only 14 patients were evaluated, and the incidence of long-term recurrence and cardiac function are unknown. Furthermore, one patient required reoperation. The optimal diameter of the pericardial fenestration and the incidence of adhesions were also not established.

## Conclusions

Although the TPW requires general anesthesia with single-lung ventilation, it is effective and safe in patients with PEs that are recurrent or difficult to drain percutaneously. Further clinical studies, especially randomized trials, are needed to evaluate the role of the TPW in such cases.

## Abbreviations

CT, Computed tomography; CVP, Central venous pressure; PE, Pericardial effusion; TPW, Thoracoscopic pericardial window; TTE, Transthoracic echocardiography

## References

[1] Muhammad M (2011). The pericardial window: is a video-assisted thoracoscopy approach better than a surgical approach?. Interact Cardiovasc Thorac Surg.

[2] O’Brien PK, Kucharczuk JC, Marshall MB, Friedberg JS, Chen Z, Kaiser LR (2005). Comparative study of subxiphoid versus video-thoracoscopic pericardial “window.”. Ann Thorac Surg.

[3] Imazio M, Adler Y (2013). Management of pericardial effusion. Eur Heart J.

[4] Cantó A, Guijarro R, Arnau A, Fernández-Centeno A, Ciscar M, Galbis J (1993). Thoracoscopic pericardial fenestration: diagnostic and therapeutic aspects. Thorax.

[5] Geissbühler K, Leiser A, Fuhrer J, Ris HB (1998). Video-assisted thoracoscopic pericardial fenestration for loculated or recurrent effusions. Eur J Cardiothorac Surg.

[6] Wong B, Murphy J, Chang CJ, Hassenein K, Dunn M (1979). The risk of pericardiocentesis. Am J Cardiol.

[7] Imazio M, Spodick DH, Brucato A, Trinchero R, Adler Y (2010). Controversial issues in the management of pericardial diseases. Circulation.

[8] Tsang TS, Barnes ME, Gersh BJ, Bailey KR, Seward JB (2003). Outcomes of clinically significant idiopathic pericardial effusion requiring intervention. Am J Cardiol.

[9] Celik S, Celik M, Aydemir B, Tanrikulu H, Okay T, Tanrikulu N (2012). Surgical properties and survival of a pericardial window via left minithoracotomy for benign and malignant pericardial tamponade in cancer patients. World J Surg Oncol.

[10] Robles R, Piñero A, Luján JA, Fernández JA, Torralba JA, Acosta F (1997). Thoracoscopic partial pericardiectomy in the diagnosis and management of pericardial effusion. Surg Endosc.

[11] Van Trigt P, Douglas J, Smith PK, Campbell PT, Wall TC, Kenney RT (1993). A prospective trial of subxiphoid pericardiotomy in the diagnosis and treatment of large pericardial effusion: a follow-up report. Ann Surg.

[12] Allen KB, Faber LP, Warren WH, Shaar CJ (1999). Pericardial effusion: subxiphoid pericardiostomy versus percutaneous catheter drainage. Ann Thorac Surg.

[13] Alcan KE, Zabetakis PM, Marino ND, Franzone AJ, Michelis MF, Bruno MS (1982). Management of acute cardiac tamponade by subxiphoid pericardiotomy. JAMA.

[14] Rocco G (2012). One-port (uniportal) video-assisted thoracic surgical resections: a clear advance. J Thorac Cardiovasc Surg.

[15] Alar T, Ozcelik C (2013). Single-incision thoracoscopic surgery of pleural effusions for diagnosis and treatment. Surg Endosc.

